# Fibronectin promotes tumor angiogenesis and progression of non-small-cell lung cancer by elevating WISP3 expression via FAK/MAPK/ HIF-1α axis and activating wnt signaling pathway

**DOI:** 10.1186/s40164-023-00419-w

**Published:** 2023-07-19

**Authors:** Fei Zhou, Jianguo Sun, Lingyun Ye, Tao Jiang, Wei Li, Chunxia Su, Shengxiang Ren, Fengying Wu, Caicun Zhou, Guanghui Gao

**Affiliations:** 1 Department of Oncology, Shanghai Pulmonary Hospital & Thoracic Cancer Institute, Tongji University School of Medicine, Shanghai, China; 2 Precision Medicine Center, Taizhou Central Hospital (Taizhou University Hospital), Taizhou, Zhejiang P R China

**Keywords:** Fibronectin, NSCLC, Metastasis, Angiogenesis, WISP3, MAPK/ERK, Wnt

## Abstract

**Background:**

Fibronectin, an extracellular matrix protein, has been reported to be associated with heterogeneous cancer stemness, angiogenesis and progression in multiple cancer types. However, the roles and the underlying mechanism of fibronectin on the progression NSCLC need to be further elucidated.

**Methods:**

Public dataset such as Kaplan-Meier Plotter was used to determine the prognostic significance of genes. The correlation of different protein expression in clinical and xenograft tissues was tested by immunohistochemistry experiment. Both in vitro and in vivo experiments were performed to determine the role of fibronectin on the tumor growth, metastasis, and angiogenesis in NSCLC. The activation of key signaling pathway under fibronectin was examined by WB assay. RNA-seq was applicated to screening the target gene of fibronectin. Rescue experiment was performed to confirm the role of target gene in fibronectin-mediated function in NSCLC. Finally, luciferase and CHIP assays were used to elucidate the mechanism by which fibronectin regulated the target gene.

**Results:**

Our results revealed that fibronectin was up-regulated in cancer tissues compared with the normal ones in NSCLC patients. Dish- coated fibronectin enhanced the tumor growth, metastasis, and angiogenesis of NSCLC in vitro and in vivo by promoting EMT and maintaining stemness of NSCLC cells. As expected, fibronectin activated FAK and its downstream MAPK/ERK signaling pathway. WISP3 was screened as a potential target gene of fibronectin. Interestingly, WISP3 effectively activated Wnt signaling pathway, and knockdown of WISP3 effectively blocked the influence of fibronectin on the migration, invasion and vascular structure formation potential of NSCLC cells. Our data also manifested that fibronectin elevated the transcription of WISP3 gene by promoting the binding of HIF-1α to the promoter region of WISP3 in NSCLC cells.

**Conclusions:**

Our findings sketched the outline of the route for fibronectin exert its role in NSCLC, in which fibronectin activated downstream FAK and MAPK/ERK signaling pathways, and mediated the accumulation of HIF-1α. Then, HIF-1α enabled the transcription of WISP3, and subsequently promoted the activation of Wnt signaling pathway, and finally enhanced the tumor growth, metastasis, and angiogenesis in NSCLC.

**Supplementary Information:**

The online version contains supplementary material available at 10.1186/s40164-023-00419-w.

## Background

NSCLC, accounting for 85% of lung cancer cases, is the almost common cause of cancer-related deaths in the world [1]. NSCLC is usually advanced at the time of diagnosis, which almost has a great metastatic potential, resulting to the low survival rate [1–3]. Presently, the treatments of NSCLC are various, including surgery, radiotherapy, chemotherapy, and immunotherapy [4, 5]. Despite the current therapy has effectively advanced the prognosis of NSCLC, metastasis of NSCLC still leads to the major rationality of death [6]. Therefore, it is indispensable to elucidate the molecular mechanisms of metastatic potential of NSCLC.

Metastasis is a multi-stage process including invasion, attachment, extravasation of vessel, angiogenesis and clone in distant tissues [7]. Additionally, EMT also affords the earlier cancer cell with invasion and metastatic capabilities, which are universally acknowledged to influence stemness and immunosuppression [8]. Numerous previous evidences illuminated that EMT might be induced by fibronectin, one of the major parts of the extracellular matrix [9–12]. Fibronectin is a glycoprotein with a 230–270 kDa size and dimer is its existence. There are almost 20 different variant in human despite of the single origination [13]. Previous studies indicated that fibronectin can be subdivided into plasma fibronectin and cellular fibronectin, the former one is always synthesized by hepatocytes and the latter one is produced by a variety of cells, such as chondrocytes and so on [14]. Furthermore, fibronectin is well-documented for its function in several biological procedures containing cell adhesion, migration, stemness, differentiation and angiogenesis [15–18]. A lot of studies have indicated that cell adhesion to extracellular fibronectin played a key role in tumorigenesis[19], and malignant progression in multiple cancer types, such as the development of the resistance to anoikis and chemotherapy [20, 21], cancer metastasis [22], and so on. However, the molecular mechanism of Fibronectin on cell proliferation, migration, invasion, angiogenesis and stemness of NSCLC remains to be enunciated.

WNT-inducible signaling pathway protein-3 (WISP-3), also named as CCN6 (Cyr61, CTGF, Nov), belongs to the CCN family [23]. CCNs, a class of cysteine-rich protein, are critical regulator components of ECM and function as signaling molecules involved in notable biological procedures, such as adhesion, invasion and angiogenesis [23–25]. In addition, a growing number of evidences have certificated that CCN6 acts a key role in carcinogenesis and metastasis [26, 27]. However, it remains to be interpreted that whether Fibronectin elevates WISP-3 expression to enhance cell proliferation, migration, invasion, angiogenesis and stemness in NSCLC.

In the present study, we investigated the expression and biological roles of fibronectin in NSCLC. We found that fibronectin was up-regulated in cancer tissues of NSCLC patients and higher fibronectin expression related with the poor prognosis. Meanwhile, fibronectin enhanced cell proliferation, colony formation, migration, invasion, and angiogenesis, maintained stemness of NSCLC probably by activating FAK and MAPK/ERK signaling pathways, and elevating the HIF-1α. Then, activated HIF-1α bound to WISP3 promoter region, triggering the transcription of WISP3 gene, which reinforced WNT/β-catenin signaling pathway and subsequently mediated metastasis and angiogenesis of NSCLC. Additionally, the knockdown of WISP3 effectively blocked fibronectin-induced cell migration, invasion, angiogenesis and cancer cell stemness. Collectively, dysregulation of fibronectin/WISP3 axis drove the progression of NSCLC, which could be the potential therapeutic target.

## Materials and methods

### Data analysis from datasets

The correlations between the overall survival and WISP3 in lung cancer was analyzed using Kaplan-Meier Plotter database.

### Human clinical specimens

The cancer tissue sections of eighteen NSCLC patients were collected from the Shanghai Pulmonary Hospital & Thoracic Cancer Institute, Tongji University School of Medicine. All patients provided written consent before enrollment in research, and the project was approved by the Institutional Ethics Committee of the Hospital and was conducted in accordance with the Declaration of Helsinki.

### Immunohistochemistry

The specimens were fixed and cut into 4-µm-thick slices. After deparaffinized and rehydrated, the sections were blocked with 3% hydrogen peroxide. Antigen retrieval on sections was performed using a steamer at 95℃. Moreover, the primary antibodies against fibronectin (dilute ratio: 1:300), WISP3 (dilute ratio: 1:200), or CD31 (dilute ratio: 1:150) were diluted and co-incubated overnight at 4 °C. Afterwards, the secondary antibodies were then added for 60 min at room temperature. Eventually, Immunohistochemical analysis was fulfilled and visualized. Representative views were obtained under a microscope (Olympus Corp., Tokyo, Japan).

### Cell culture

The NSCLC cell lines, H460, H1299, HCC827, A549 as well as H1975 were procured from ATCC (American Type Culture Collection, Rockville, MD, USA). All cell lines were cultured in DMEM supplemented with 10% FBS, penicillin (100 U/mL) and streptomycin (100 µg/mL) in a humidified incubator with an atmosphere of 5% (v/v) CO_2_ at 37℃. The culture medium was replaced every two days. The cells in the logarithmic growth phase were collected for the follow-up experiments.

Soluble fibronectin, purified from human plasma, used in this study was obtained from Sigma (F2006). Fibronectin was initially dissolved using sterile water as a stock at concentration of 1 mg/ml, and then diluted using PBS to 10 µg/mL as a working solution. Dishes were coated with fibronectin working solution or PBS, and dried at room temperature for 45 min. In all cellular experiments, NSCLC cells were pre-cultured in control or fibronectin-coated dishes for 24 h.

### Transwell assays

The invasion and migration assay were performed in a Transwell chamber (Corning) precoated with or without Matrigel (BD Biosciences). After seeded with cells suspension in serum-free DMEM medium, the lower chambers were then fulfilled with DMEM contained 10% FBS as a chemoattractant. After 24 h incubation, cells in the upper chamber were removed and the membrane was washed three with PBS. The cells attracted to the lower surface of the insert were fixed, then stained using 0.5% crystal violet and counted under a microscope (Olympus Corp., Tokyo, Japan). The experiment was performed at least three times independently.

### Wound healing assay

Cell migration was also confirmed by wound healing experiment. In brief, cells were cultured in dishes coated with or without 10 µg/mL fibronectin for 24 h, and then seeded into six-well plate (10^4 cells/well). After cells attached overnight, a pipette tip was used to create an incision-like gap, and cell migration is quantified and expressed as average percentage of closure of the scratch area at 24 and 48 h.

### Cell proliferation

Cell proliferation was assessed using MTT assay in medium with fibronectin-free serum which was obtained after absorbing FN in FBS with excess gelatin beads (BSZH company, Beijing, China). After incubation in control or 10 µg/mL fibronectin-coated dish for 24 h, H460 and H1299 cells were then seeded into 96-well plates (5000 cells/well) and incubated for another 24 h, 48 h, 72 h, and 96 h. After that, the cells were added 50 µl MTT solution (0.5 mg/ml in PBS) for 2-4 h, and then the formed purple crystal was dissolved by 150 µl DMSO. The Microplate Reader (Molecular Device) was used to detect the signal by reading the absorbance at 570 nm. The experiment was performed at least three times independently.

### Colony formation

After incubation in control or 10 µg/mL fibronectin-coated dish for 24 h, cells were seeded at a density of 300 cells/well into 6-well plates, and followed by another 14 days incubation. After that, cells were then washed with PBS. Then, colonies were fixed with methanol and stained by crystal violet solution. Eventually, the number of colonies (> 50 cells) was enumerated. The survival fractions were the ratio of the treatment groups to the untreated controls. The experiment was performed at least three times independently.

### Xenograft experiment for tumor growth detection

5 × 10^6^ H1299 cells were suspended in 0.2 ml saline mixed with or without fibronectin (10 µg/mL) for 30 min, and subcutaneously injected into Balb/c nude mice (female, 4 weeks old, 20 g; Vital River, China) to generate xenograft model, and each group contains six mice. All animal studies were conducted in compliance with the regulations and guidelines of Tongji University institutional animal care, and according to the AAALAC and the IACUC guidelines. Three weeks later, all tumors were removed from mice, and the tumor size was measured by a vernier caliper and the tumor volume was calculated by the volume formula of hemiellipsoid (volume = length × width × height/2).

### RNA sequencing

Total RNA of H460 cells cultured in dishes coated with or without 10 µg/mL fibronectin for 24 h were extracted by Trizol reagent (Invitrogen). RNA-Sequencing experiments were performed by LC-Bio Technology CO., Ltd. (Hangzhou, China) based on the illumina Novaseq™ 6000 system. Gens with more than two-fold difference in expression between control groups and fibronectin-treated groups were selected as differentially expressed genes (DEGs). GO analysis on DEGs was implemented with an R package. Pathway analysis was determined by the significant pathway of the differential genes according to Kyoto Encyclopedia of Genes and Genomes Database. P value < 0.05 as a statistically significant.

### qRT-PCR

Total RNA of H460 cells cultured in dishes coated with or without 10 µg/mL fibronectin for 24 h were extracted by Trizol reagent (Invitrogen). cDNA was synthesized using a cDNA Synthesis Kit (Promega). Real-time PCR was performed by ABI 7300 Fast Real-Time PCR system (Applied Bioscience, Foster City, CA) according to manufacturer’s protocol. The PCR procedure included initial denaturation at 95˚C for 10 min, followed by 40 cycles of denaturation at 95˚C for 15 s, annealing and extension at 60˚C for 1 min. The Ct values were acquired and contrasted using the 2^−ΔΔCt^ method. β-actin was used as a normalized control. The primers sequences used were listed in Table 1.

**Table 1:** Sequences of primers in qPCR experiments

Gene names	F(5’-3’)	R(5’-3’)
β-actin	AGCAGTTGTAGCTACCCGCCCA	GGCGGGCACGTTGAAGGTCT
CPA4	CCTGGTCCCATCTGTCA	CCATCTCGTGGTAAATAGC
PLCB4	AAGGCAAGGAAGGACAGGTG	CGTTGTTGGCCCTGAAGTTG
EDN1	CTCTGCTGTTTGTGGCTTGC	CTCGGGAGTGTTGACCCAAA
KIF27	GGTGTCAGCCAAACTACCCA	CAGCCTTCCTGACCTCTTGG
RGPD5	GAAAGGGGCTTGGGGAACTT	TGTCTAACAGAAGCCGCTGG
ANKRD1	CTTCTAGCCCACCCTGTGAC	GCACATCCACAGGTTCCGTA
PTX3	AAGCGTGCATCCAGTGAGAC	TACCCACAAGGATGTGAGCC
WISP3	GGTGGCTCCTTATTCCCACT	TCGCAGTAGAGACCCTTGTG
THBS1	GGAGGAGGGGTACAGAAACG	GGGACAAGCACCACATTTCC

### Western blotting

RIPA lysis buffer containing 1% protease inhibitor cocktail (Sigma) was used for protein extraction. After electrophoresed on 8–15% SDS-PAGE gels, proteins were transferred to PVDF membranes (Millipore), and blocked with 5% skim milk for 2 h. Then, the membrane was incubated with the primary antibodies against fibronectin (1:1000, Proteintech, 15613-1-AP), WISP3(1:800, Proteintech, 21259-1-AP), p-FAK (1:1000, abcam,ab81298), FAK (1:1000, Proteintech, 66258-1-Ig), p-MEK (1:1000, CST, 9154 S), MEK (1:1000, proteintech, 11049-1-AP), p-ERK (1:1000, CST, 4370 S), ERK (1:1000–1:2000,ABclonal, A16686), p-β-catenin (1:1000, proteintech, 28772-1-AP), β-catenin (1:1000, proteintech, 51067-2 -AP), N-cadherin (1:500, proteintech, 22018-1-AP), vimentin (1:1000, proteintech, 10366-1-AP), OCT4 ( 1:1000-1:5000, proteintech, 11263-1-AP), Nanog (1:1000, CST, 4903s), VEGF (1:1000, ABclonal, A12303), CD31 (1:500, Proteintech, A0378), SOX2 (1:1000, CST, #14,962), Tie2 (1:500, Proteintech, 19157-1-AP), Ve-cadherin (1:1000, Proteintech, 66804-1-Ig), and HIF-1a (1:2000, Proteintech, 20960-1-AP)at 4℃ overnight, β-actin was used as a loading control. The corresponding horseradish peroxidase-conjoined secondary antibody was then applied for 1 h at RT. After chemiluminescence reaction with HRP substrate, signals were visualized by Pierce®ECL Plus kit using a ChemiDoc XPS system (Bio-Rad Laboratories, Inc., Hercules, CA, USA).

### 3D tube formation

After culture in dishes coated with or without 10 µg/mL fibronectin for 24 h, cell suspensions were prepared and placed on the surface of Matrigel (BD Biosciences, Franklin Lakes, NJ, USA) in 4-well chamber slides (BD Biosciences) at the density of 50 µl/well. Reorganization of cells and the formation of capillary-like structures were monitored at 0.5-2 h.

### Luciferase reporter assay

For luciferase assays, the human WISP3 mRNA, containing the HIF-1α binding site and corresponding mutant sequence, were amplified and cloned into the pGL3 vector. Then pGL3 empty vector or pGL3- WISP3 (wild type or mutant) were transfected into H460 and H1299 cells and the transfected cells were seeded in 24-well plates and treated with control or 10 µg/mL fibronectin for 24 h. After that, cells were harvested and lysed and the luciferase activity was determined by Dual-Glo Luciferase Assay System (Promega) according to manufacturer’s instructions. Luciferase activity was standardized to Renilla luciferase activity.

### RNA interference, plasmid construction and transfection

The siRNAs against WIPS3 and HIF-1α were purchased from GemmaPharma (Shanghai, China). Cells were transfected with siRNA using Lipofectamine 2000 (Invitrogen) according to manufacturer’s instructions. A non-specific scramble siRNA sequence was used as negative control (NC). The sequence of siR-WISP3 is 5’-CCAGGGGAAAUCUGCAAUG-3’ and the sequence of siR-HIF-1α is 5’-AAAGGACAAGUCACCACAGGA-3’, the sequence of siR-NC is 5’-UUCUCCGAACGUGUCACGUTT − 3’.

The WISP3 overexpression plasmid was constructed based on PCDH vector provided by Youbao Bio. (Changsha, China). The plasmid was incubated with cells using Lipofectamine 2000 (Invitrogen) for 20 min at room temperature to form complexes, and transient transfection was executed according to manufacturer’s instructions.

### Chromatin immunoprecipitation (CHIP) assay

Chromatin immunoprecipitation (ChIP) assays were performed using antibody against HIF-1α (Cell Signaling Technology) and a ChIP kit (Cell Signaling Technology) according to manufacturer’s protocols. The DNA fragments of WISP3 promoter with different HIF-1α potential binding sites in the CHIP product was measured by qPCR. The PCR primer sequences for three HIF-1α binding sites are as follows: site 1: 5’- TCTAGGCTGCTTCCTTCTCC-3’ (forward), 5’- GCTGCGATGGGTGCTGTTG-3’ (reverse); site 2: 5’- CCTGTTCCTGGAGAGCAGAGCC TGC-3’ (forward), 5’- GTGAAGCTGGAGAAGGAAGCAGCCT-3’ (reverse); site 3: 5’- CTG CCTTCGCAGTGAGCGTGTCACA − 3’ (forward), 5’- CCCCACCCGTTGTCGGGGGCAGGCT-3’ (reverse).

### Statistical analysis

Statistical analysis was fulfilled with the GraphPad Prism 8.0 (GraphPad, Inc., USA) based on 3 repeat experiments at least. Representative data were shown as means ± SD. Significance analysis was performed using two-tail Student t test for two groups or one-way ANOVA for multiple groups. And P < 0.05 (*) was considered significant.

## Results

### Fibronectin promoted cancer cell growth and metastasis of NSCLC

**Fig. 1:**
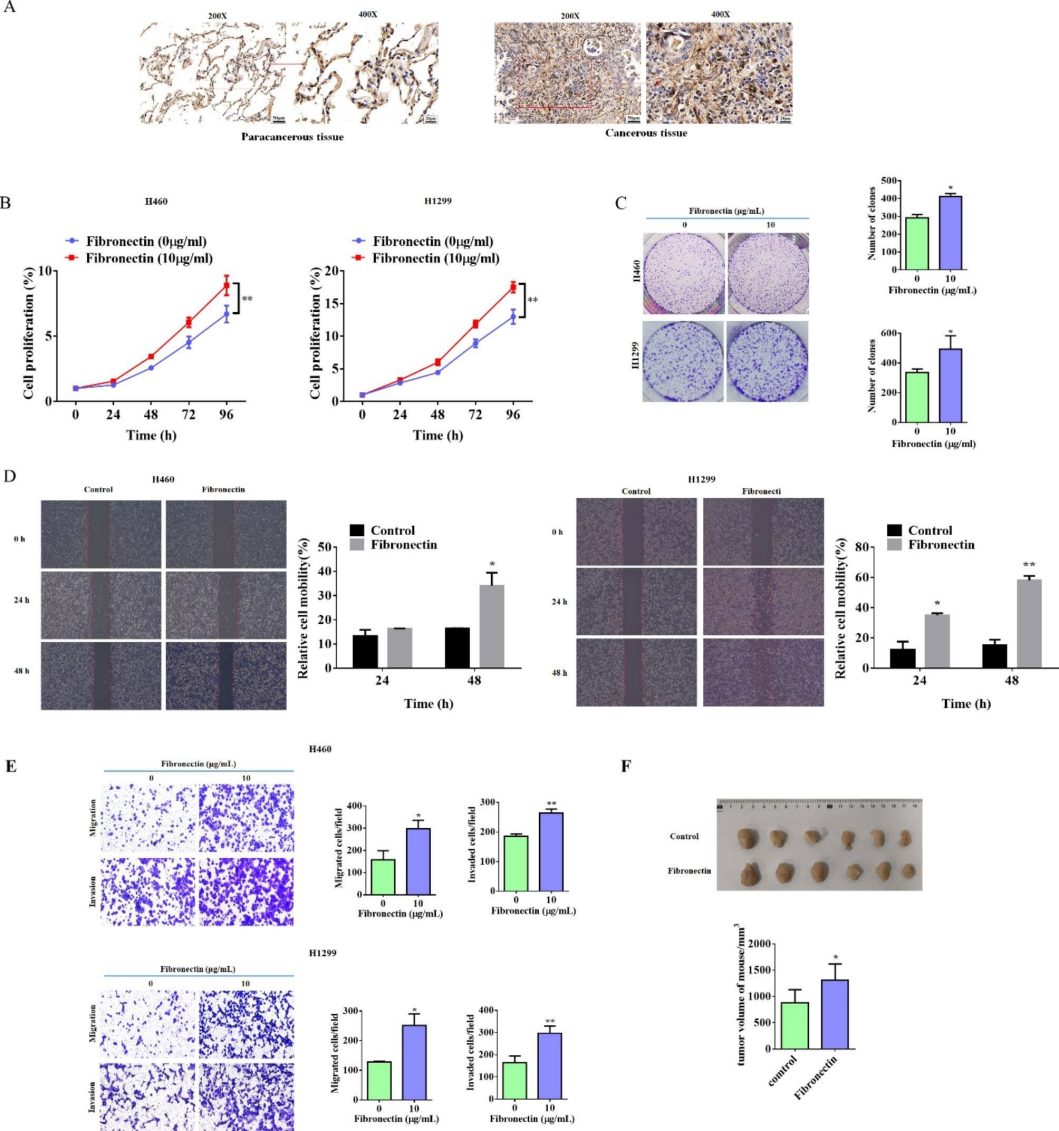
Fibronectin significantly enhanced metastasis and slightly promoted tumor growth of NSCLC. (**A**) Representative IHC staining images for Fibronectin in paracancerous tissue (P) and cancer tissues from NSCLC patients. Brown color displays Fibronectin protein levels, with counterstaining by hematoxylin in blue. (**B**) The proliferation abilities of H460 and H1299 cells treated with or without dish-coated fibronectin (10 µg/mL) for 24 h were measured by MTT assay at the indicated time points. (**C**) Representative photographs of colony formation in H460 and H1299 cells that treated with or without dish-coated fibronectin (10 µg/mL) for 24 h. The number of colonies was photographed and analyzed in the histogram. (**D**) The migration abilities of H460 and H1299 cells treated with or without dish-coated fibronectin (10 µg/mL) for 24 h were detected by wound healing assay at 24 and 48 h. (**E**) The migration and invasion abilities of H460 and H1299 cells treated with or without dish-coated fibronectin (10 µg/mL) for 24 h were detected by Transwell assay. Quantitative analysis of migrated and invaded cells was shown in the histogram. (**F**) 5 × 10^6^ H1299 cells were suspended in 0.2 ml saline with or without fibronectin (10 µg/mL), and subcutaneously injected into nude mice to generate xenograft model. Three weeks later, all tumors were removed from mice and listed for the photo. Data were presented by mean ± SD for cell experiment, or mean ± SEM animal experiments from three independent experiments. *P < 0.05; **P < 0.01 vs. control

As an ECM protein, previous studies have manifested the crucial role of fibronectin in cancer progression in multiple cancer types including lung cancer, [28–30]. However, the underlying mechanisms still remains elusive. The IHC assay in NSCLC demonstrated that a higher fibronectin protein expression was observed in cancer tissues compared with the paracancerous ones (Fig. 1A). To initially confirm the pro-tumor function of fibronectin in NSCLC, we tested its roles on cell proliferation in several NSCLC cell lines, such as HCC827, A549, H1975, H460, and H1299 cells. Our data indicated that dish-coated fibronectin treatment effectively promoted the cell proliferation of H460 and H1299 (Fig. 1B). Similarly, fibronectin mildly promoted the colony formation activities of the above two cell lines with statistical significance (Fig. 1C). Moreover, only the potentials of cell migration and invasion of HCC827, H460, and H1299 cells were significantly enhanced by fibronectin, while an inhibitory function was observed in A549 and H9175 cells (Fig. 1D &E, and S Fig. 1). Next, the role of fibronectin on tumor growth in vivo was examined using a xenograft model, which was established by subcutaneous injection. Then the H1299 cells were suspended with or without fibronectin (10 µg/mL) in saline. Our data revealed that fibronectin also slightly but significantly promoted the growth of H1299-derived xenografts (Fig. 1F). Thus, these data supported that fibronectin could effectively promote tumor growth and metastasis of NSCLC both in vitro and in vivo.

### RNA-seq identified WISP3 as a potential target gene of fibronectin

**Fig. 2:**
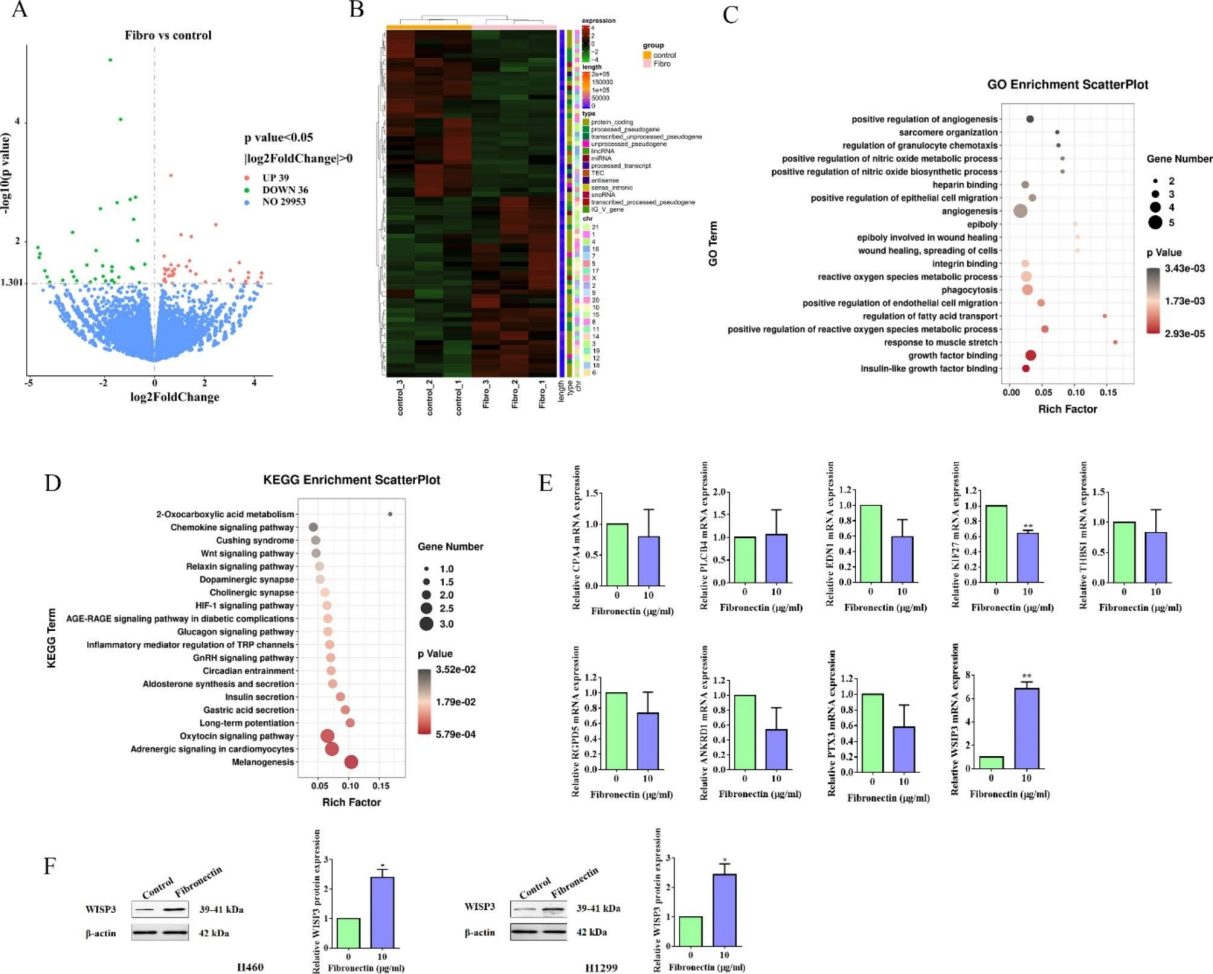
WISP3 was identified as the candidate target of Fibronectin. (**A**) Volcano plots, showed genes with partial expression in the control and the dish-coated fibronectin-treated groups in H1299 cells. Red dots showed genes with dramatically higher abundances while the green dots represented genes with significantly lower levels of expression. Values on the x-axis was log2 fold change (log2FC), and the y-axis was log10 p value (log10pvalue). (**B**) Heat-map of differentially expressed genes from RNA-seq of the control and the fibronectin treated groups. (C and D) Functional analyzing by GO (**C**) and KEGG (**D**) of differentially expressed genes in control groups and fibronectin-treated groups. (**E**) The top 9 differentially expressed genes were subject to qPCR for validation. (**F**) Western blotting analysis of WISP3 in H460 and H1299 cells treated with or without dish-coated fibronectin (10 µg/mL). Data were presented by mean ± SD from three independent experiments. *P < 0.05; **P < 0.01 vs. control

To seek out the unambiguous mechanisms underpinning the inspected Fibronectin-dependent phenotypes, RNA-seq was utilized in dish-coated fibronectin-treated and control H460 cells. As illustrated in the Volcano plot, RNA-seq identified 39 differentially expressed genes (DEGs) that were up-regulated after fibronectin treatment, while 36 were down-regulated. (Fig. 2A). Based on those DEGs, the heatmap (Fig. 2B) and enrichment analysis (Fig. 2C&D) were generated, one can perceive that, GO enrichment analysis indicated that angiogenesis, and growth factor binding was enriched (Fig. 2C). In addition, KEGG enrichment analysis obtained several enriched signaling pathways included Wnt, HIF-1, relaxin, glucagon, GnRH, and oxytocin signaling pathways (Fig. 2D). Moreover, we then selected top 9 candidate DEGs including CPA4, PLCB4, EDN1, KIF27, THBS1, RGPD5, ANKRD1, PTX3, WISP3 for qPCR validation. Interesting enough, only KIF27 was significantly down-regulated among them, while WISP3 was effectively up-regulated by fibronectin treatment (Fig. 2E). Intriguingly, the data from WB assay shown that the protein expression of WISP3 was invariably discovered to be up-regulated by dish-coated fibronectin treatment in both H460 and H1299 cells (Fig. 2F). Taken all together, WISP3 may be the target of Fibronectin.

### Fibronectin activated FAK, WNT/β-catenin, MAPK/ERK signaling pathways

**Fig. 3:**
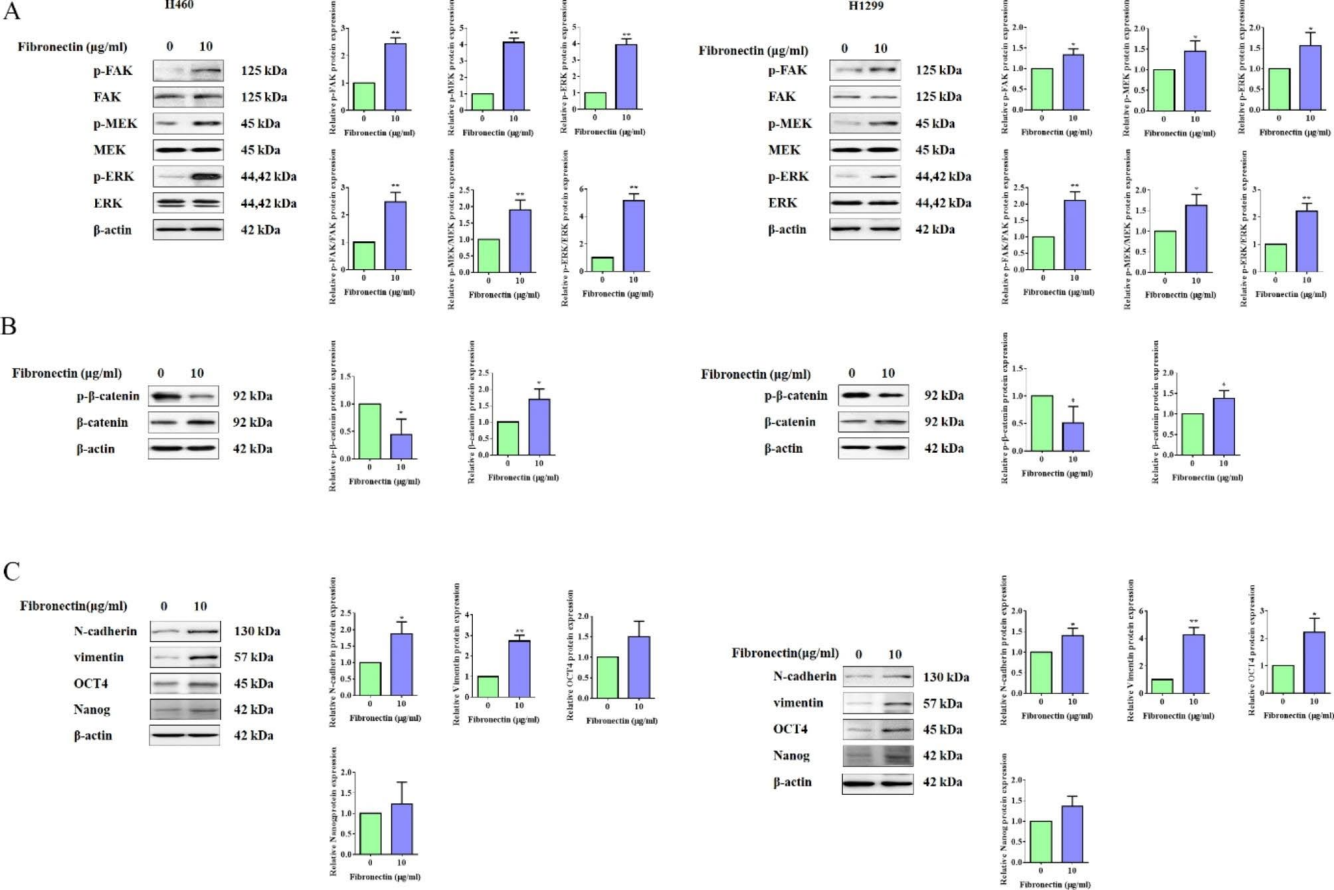
Fibronectin activated FAK, WNT/β-catenin, MAPK/ERK signaling pathways, and promoted EMT and stem cell properties in NSCLC cells. (**A**) H460 and H1299 cells were treated with or without dish-coated fibronectin (10 µg/mL) for 24 h. Then, Western blot was performed for the detection the proteins of p-FAK, FAK, p-MEK, MEK, p-ERK and ERK. β-actin was used as a loading control. Quantitative analysis of phosphorylated proteins expression were shown in the histograms. (**B**) H460 and H1299 cells were treated with or without dish-coated fibronectin (10 µg/mL) for 24 h. Then, t Western blot was performed for the detection the proteins of p-β-catenin and β-catenin. β-actin was used as a loading control. Quantitative analysis of p-β-catenin and β-catenin expression were shown in the histogram. (**C**) H460 and H1299 cells were treated with or without dish-coated fibronectin (10 µg/mL) for 24 h. Then, Western blot was performed for the detection the proteins of N-cadherin, vimentin, OCT4 and Nanog. β-actin was used as a loading control. Quantitative analysis of proteins expression were shown in the histogram. Data were presented by mean ± SD from three independent experiments. *P < 0.05; **P < 0.01 vs. control

It is well known that extracellular fibronectin can directly bind to integrin, which located on the cell surface, to activated downstream signaling pathways such as FAK and MAPK/ERK [31, 32]. Here, we examined the activation of FAK and MAPK/ERK signaling pathways in both H460 and H1299 cells under dish-coated fibronectin treatment. As shown in Fig. 3A, elevated phosphorylation of FAK, MEK and ERK in both two NSCLC cell lines with dish-coated fibronectin treatment were observed, which was consistent with the previous publication. Our data from KEGG enrichment analysis also suggested Wnt signaling pathway was enriched. Therefore, we next detected the activation of Wnt signaling pathway in H460 and H1299 cells treated with dish-coated fibronectin. As shown in Fig. 3B, fibronectin treatment effectively decreased the phosphorylation of β-catenin, while elevated the expression of β-catenin protein in both two cell lines (Fig. 3B), meaning the activation of Wnt signaling pathway. Previous studies have revealed that the activation of FAK, Wnt/β-catenin and MAPK/ERK pathways in inducing cancer cell migration, invasion and EMT [33–35]. So we next determined the EMT of NSCLC cells under fibronectin, and as shown in Fig. 3C, fibronectin treatment promoted expression of EMT-related proteins including N-cadherin and vimentin both in two cell liens. Due to the critical regulation role of Wnt signaling pathway on stem cell properties [36], the expression levels of related biomarkers of stem cell including OCT4 and Nanog were examined by WB experiment. The results showed that dish-coatedFibronectin clearly increased the expression of both OCT4 and Nanog (Fig. 3C).

### Fibronectin could facilitate angiogenesis in NSCLC

**Fig. 4:**
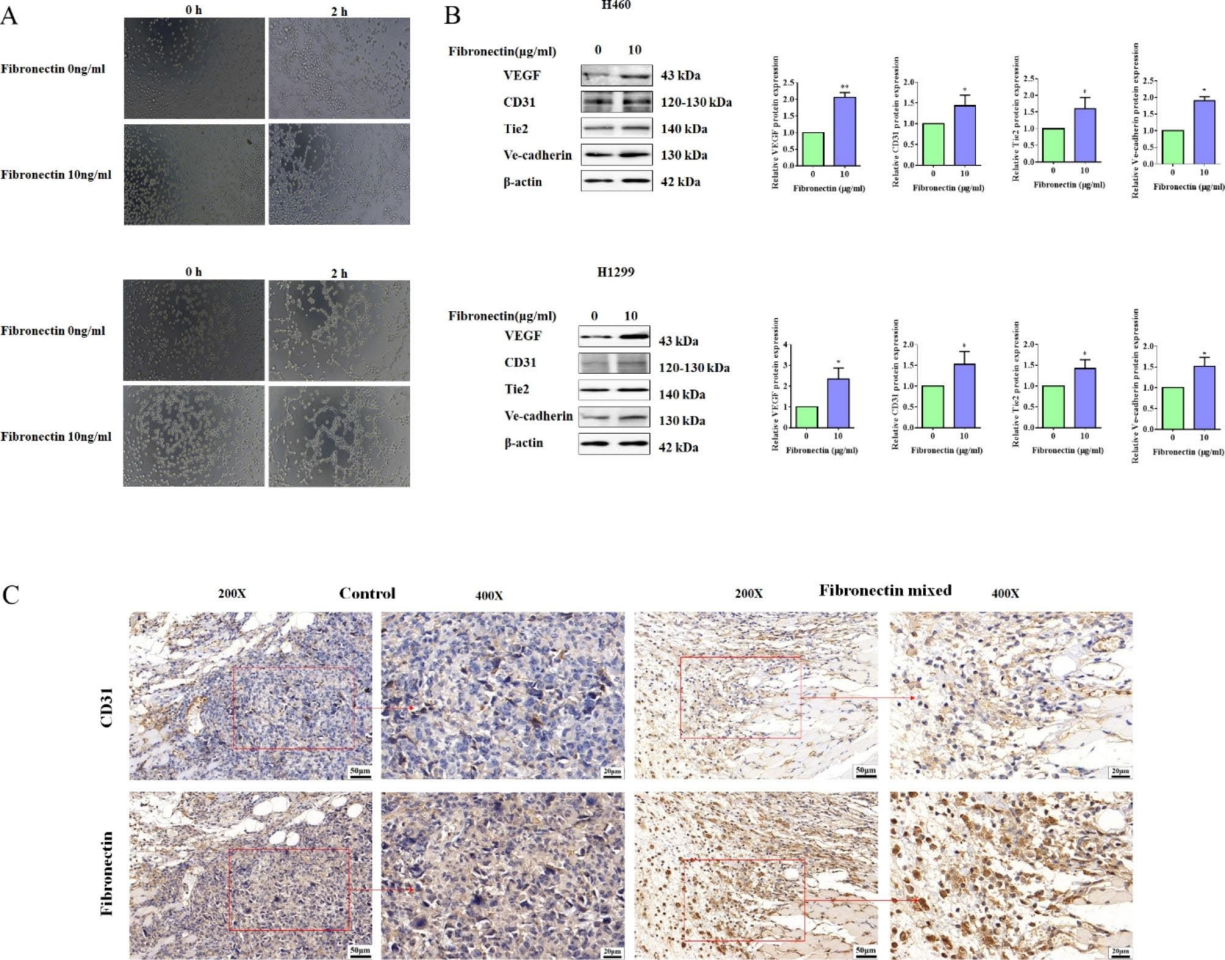
Fibronectin positively regulated the angiogenesis in NSCLC. (**A**) In vitro Matrigel assay was performed to detect the vascular structure formation in H460 and H1299 cells treated with or without dish-coated fibronectin (10 µg/mL) for 24 h. (**B**) H460 and H1299 cells were treated with or without dish-coated fibronectin (10 µg/mL) for 24 h. Then, Western blot was performed for the detection the proteins of VEGF, CD31, Tie2 and Ve-cadherin. β-actin was used as a loading control. Quantitative analysises of proteins expression were shown in the histogram. (**C**) IHC assay was used to determine the expression of fibronectin and CD31 in tumor tissues from xenograftsderived from H1299 cells mixed with or without fibronectin, and representative data were shown. Data were presented by mean ± SD from three independent experiments. *P < 0.05; **P < 0.01 vs. control

As shown above, the GO enrichment analysis suggested that fibronectin might take part in the regulation of angiogenesis. A growing number of evidences also well documented the indispensable influence of angiogenesis in cancer progression and metastasis [37, 38]. In vitro 3D tube formation experiment was performed to assess the role of fibronectin on angiogenesis. The results illustrated that both H460 and H1299 cells treated with dish-coated fibronectin exhibited stronger vascular structure-forming activities within 2 h incubation in contrast to the cells of control group (Fig. 4A). Further Western blotting experiments testified that dish-coated Fibronectin triggered the expression of angiogenesis-related proteins containing VEGF, CD31, Tie2, and Ve-cadherin (Fig. 4B). More importantly, IHC assay was performed in H1299 cell-derived xenografts to confirm the relationship between fibronectin and CD31 (a biomarker associated with blood vessel density). It was observed that more CD31 staining intensity was obtained in tumors concomitant with fibronectin mixture. Which implied a positive correlation between fibronectin and angiogenesis in NSCLC (Fig. 4C). Therefore, we enunciated that fibronectin triggered angiogenesis in vitro and in vivo.

### WISP3 strengthened cell migration, invasion, colony formation and angiogenesis in NSCLC cells

**Fig. 5:**
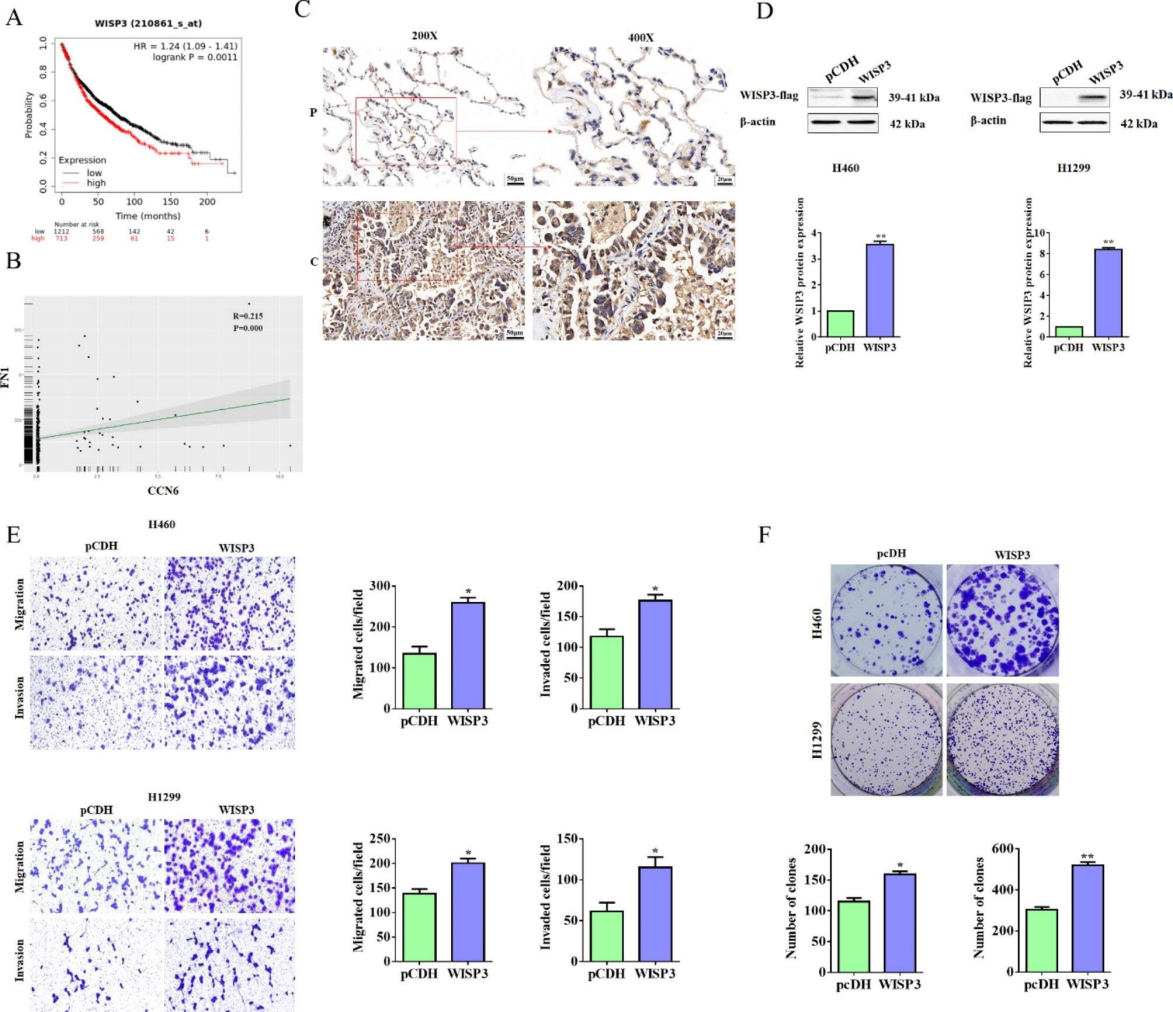
WISP3 strengthened cell migration, invasion and colony formation in NSCLC. (**A**) Correlation between expression of WISP3 and overall survival of lung cancer patients. Data were obtained from the Kaplan-Meier Plotter database. (**B**) The correlation of WISP3 (CCN6)and fibronectin (FN1) was analyzed using sequencing data from TCGA. (**C**) Representative IHC staining images for WISP3 in paracancerous tissues (P) and cancer tissues from NSCLC patients. Brown color displays Fibronectin protein levels, with counterstaining by hematoxylin in blue. (**D**) Western blot was performed to detect the overexpression of WISP3 in H460 and H1299 cells transfected with pCDH or pCDH-WISP3 for 24 h. Quantitative analysis of proteins expression were shown in the histogram. (**E**) The migration and invasion ability of H460 and H1299 cells transfected with pCDH or pCDH-WISP3 were detected by Transwell assay. Quantitative analysis of migrated and invaded cells was shown in the histogram. (**F**) Representative photographs of colony formation in H460 and H1299 cells that transfected with pCDH or pCDH-WISP3. The number of colonies was photographed and analyzed in the histogram. Data were presented by mean ± SD from three independent experiments. *P < 0.05; **P < 0.01 vs. control

As mentioned above, WISP3 levels expressed in NSCLC cells were up-regulated due to fibronectin treatment, made it a potential target gene of fibronectin. Although it has been reported that WISP3 acted as a tumor suppressor in several cancer types [39, 40], it exerted a positive function in angiogenesis [24]. On the other hand, little is known about the biological roles of WISP3 in lung cancer especially in NSCLC. In this study, we found that WISP3 mRNA levels was negatively associated with the overall survival of lung cancer patients, which was obtained by dataset of Kaplan-Meier Plotter (Fig. 5A). The analysis of sequencing data from TCGA indicated that there is a positive correlation between WISP3 and fibronectin expression in NSCLC (Fig. 5B). The expression profiling of WISP3 protein in NSCLC tissues and paracancerous ones was also detected by IHC experiment, and as shown in Fig. 5C, apparently higher level of WISP3 protein was observed in cancer tissues. All these data suggested WSIP3 might be a pro-cancer gene in NSCLC. So we constructed WISP3 overexpression plasmid and transfected into H460 and H1299 cells respectively, and both of them two had a higher WISP3 expression level (Fig. 5D). Furthermore, the results from transwell and colony formation assays revealed that overexpressing WISP3 also effectively promoted the cell migration and invasion (Fig. 5E) and colony growth activities in both H460 and H1299 cells (Fig. 5F).

**Fig. 6:**
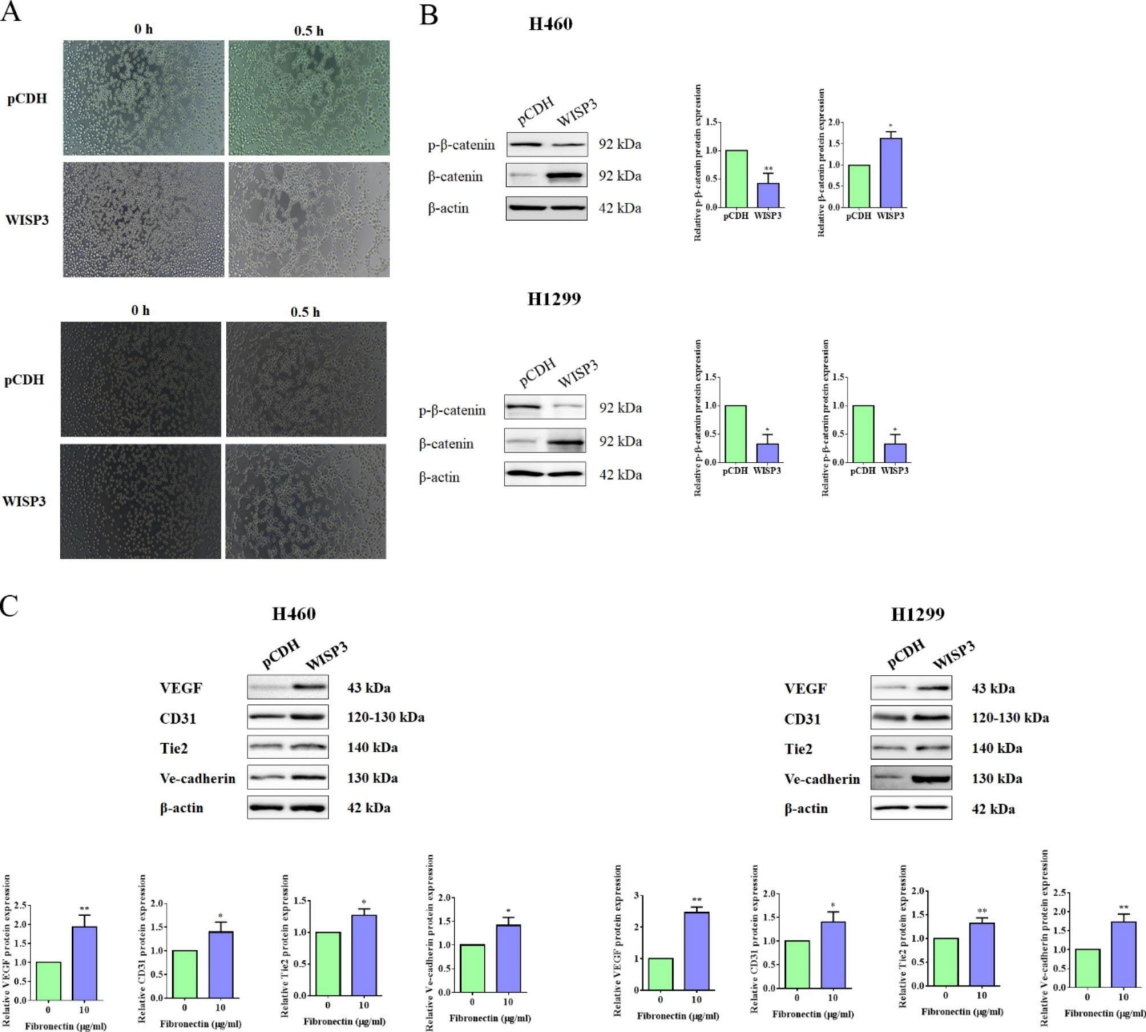
WISP3 promoted tube forming potential of NSCLC cells in vitro. (**A**) In vitro Matrigel assay was performed to detect the vascular structure formation in H460 and H1299 cells transfected WISP3 overexpression plasmid (pCDH-WISP3) or empty vector (pCDH) for 24 h. (**B**) H460 and H1299 cells were transfected with pCDH or pCDH-WISP3 for 24 h. Then, Western blot was performed for the detection the proteins of p-β-catenin and β-catenin. β-actin was used as a loading control. Quantitative analysis of p-β-catenin and β-catenin expression were shown in the histogram. (**C**) H460 and H1299 cells were transfected with pCDH or pCDH-WISP3 for 24 h. Then, Western blot was performed for the detection the proteins of VEGF, CD31, Tie2 and Ve-cadherin. β-actin was used as a loading control. Quantitative analysis of proteins expression were shown in the histogram. Data were presented by mean ± SD from three independent experiments. *P < 0.05; **P < 0.01 vs. control

Next, the vascular structure formation potential of H460 and H1299 cells with WISP3 overexpressed was also examined. As shown in Fig. 6A, cells with WISP3-overexpressedexerted stronger vascular structure formation activities compared those in the control group. As a member of WNT1 inducible signaling pathway (WISP) family, it has been reported that WISP3 might also influence the activation of Wnt signaling pathway probably as a feedback loop [41]. But, whether WISP3 could activate Wnt signaling pathway in NSCLC in turn or not is unknown. In the present study, our data revealed that WSIP3 overexpression significantly decreased the phosphorylation of β-catenin, but increased the expression of β-catenin protein in H460 and H1299 cells (Fig. 6B), provided a compelling idea that the overexpression of WSIP3 induced to the activation of Wnt signaling pathway. Furthermore, and as expected, the ectopic expression of WISP3 also effectively promoted the expression of angiogenesis-related proteins containing VEGF, CD31, Tie2, and Ve-cadherin (Fig. 6C).

### Fibronectin induced cell mobility and angiogenesis in a WISP3-dependent manner in NSCLC

**Fig. 7:**
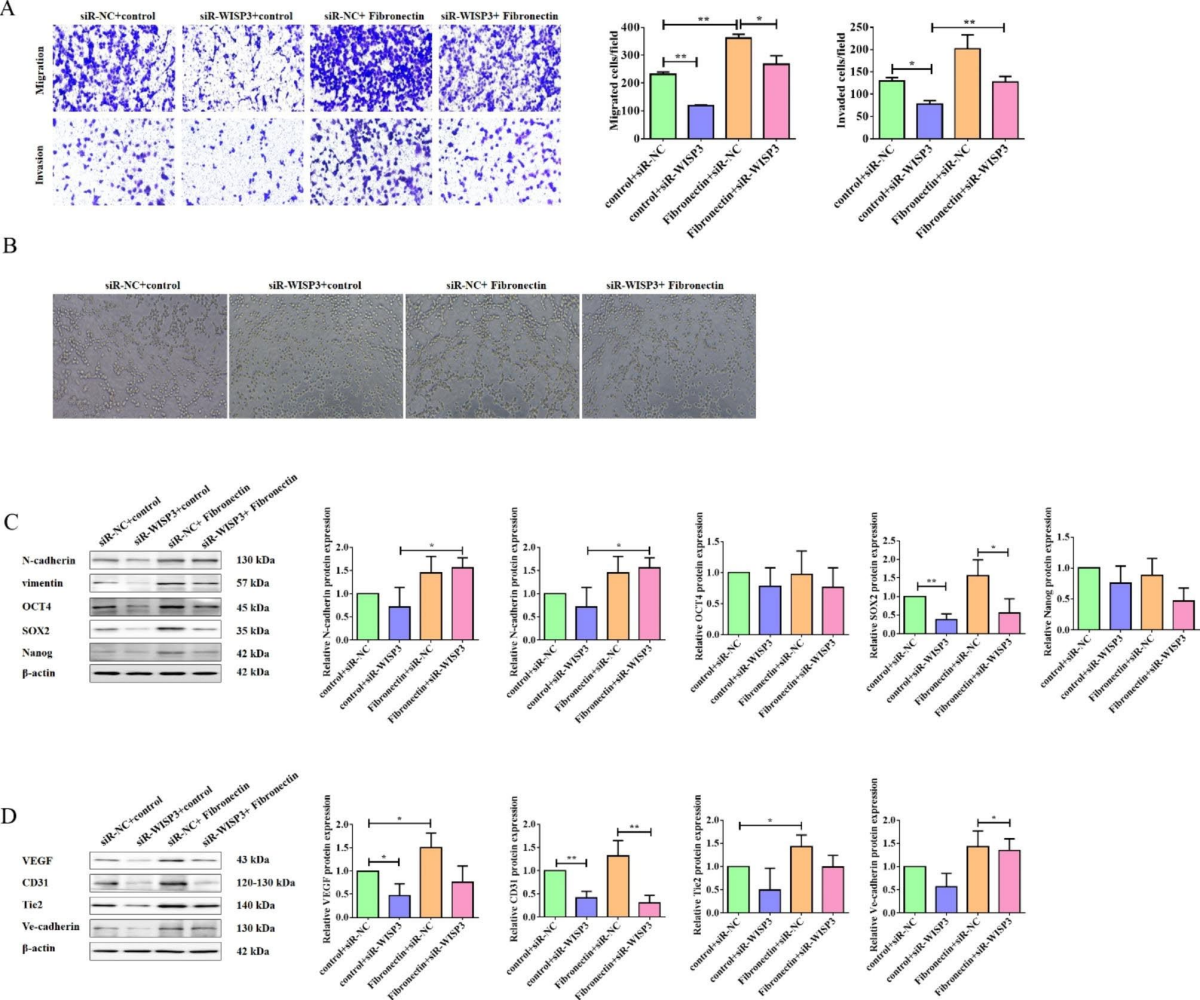
Knockdown of WISP3 blocked Fibronectin-promoted cell migration, invasion, angiogenesis and cancer stemness in NSCLC cells. (**A**) H1299 cells were transfected with siNC or siWISP3 for 24 h. Then, the migration and invasion ability of the transfected cells treated with or without dish-coated fibronectin were detected by transwell assay. Quantitative analysis of migrated and invaded cells was shown in the histogram. (**B**) In vitro Matrigel assay was performed to detect the vascular structure formation in H1299 cells treated as (**A**). (**C**) Western blot assay was used to detect the proteins of N-catenin, vimentin, OCT4, SOX2 and Nanog in H1299 cells as treated in (**A**). β-actin was used as a loading control. Quantitative analysis of proteins expression were shown in the histogram. (**D**) Western blot assay was used to detect the proteins of VEGF, CD31, Tie2 and Ve-cadherin in H1299 cells treated as (**A**). β-actin was used as a loading control. Quantitative analysis of proteins expression were shown in the histogram. Data were presented by mean ± SD from three independent experiments. *P < 0.05; **P < 0.01 vs. control

To test the functional influence of WISP3 on fibronectin-induced cell migration, invasion, and angiogenesis in NSCLC, we used siRNA to knockdown WISP3 in H1299 cells. As expected, fibronectin treatment accelerated migration and invasion of H1299 cells, while knocking WISP3 down impaired the elevated fibronectin-induced cell migration and invasion (Fig. 7A). Similar result was also obtained in the 3D tube formation assay, that is knockdown of WISP3 effectively suppressed Fibronectin-promoted vascular structure formation potential in H1299 cells (Fig. 7B). Interestingly, knockdown of WISP3 also significantly blocked the promoted fibronectin-triggered expression of EMT-related proteins including N-cadherin, vimentin, and stemness-related proteins containing OCT4, Nanog (Fig. 7C). Importantly, our data also confirmed that knockdown of WISP3 obviously inhibited Fibronectin-enhanced expression of angiogenesis-related proteins including VEGF, CD31, Tie2, and Ve-cadherin (Fig. 7D). In aggregate, our results manifested that Fibronectin could promote cell mobility, angiogenesis and stemness via elevating WISP3 expression in NSCLC.

### Fibronectin activated WISP3 expression at transcriptional level via HIF-1α in NSCLC

**Fig. 8:**
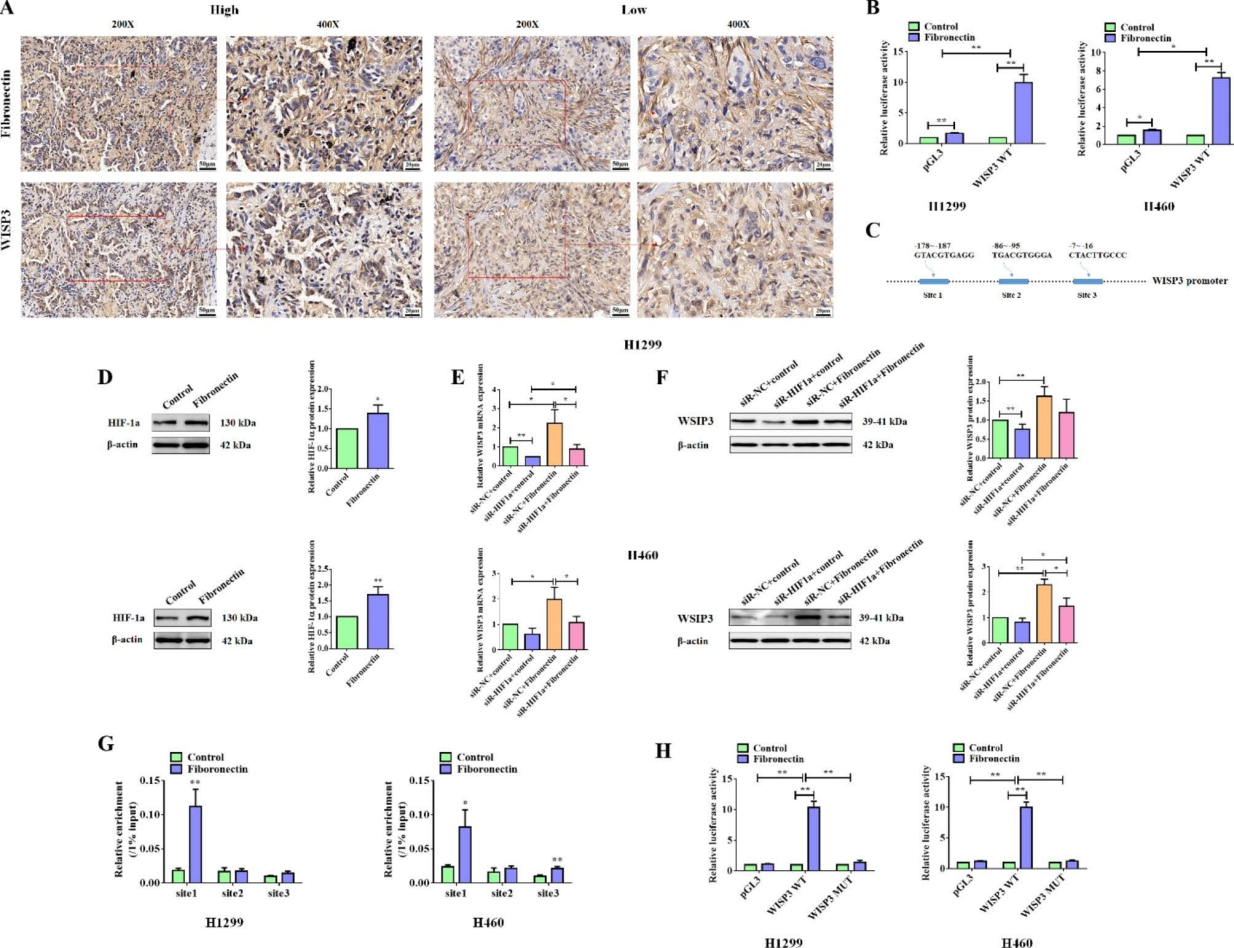
Fibronectin up-regulated WISP3 expression by activating HIF-1α in NSCLC cells. (**A**) IHC assay was performed to detect the WISP3 expression in NSCLC cancer tissues with high or low fibronectin expression, and representative data were shown. (**B**) Luciferase experiments were used to determine the role of fibronectin on the transcriptional activity of WISP3 promoter. (**C**) The potential binding site for HIF-1α to WISP3 promoter was predicted by JASPAR dataset and details were shown in the schematic diagram. (**D**) Western blot assay was used to detect the proteins of HIF-1α in H1299 cells treated with or without dish-coated fibronectin (10 µg/mL) for 24 h. β-actin was used as a loading control. Quantitative analysis of proteins expression were shown in the histogram. (**E**) H1299 and H460 cells were transfected with siRNA against HIF-1α or negative control (NC) for 24 h, followed by treating with or without dish-coated fibronectin (10 µg/mL) for 24 h. Then, qPCR was performed to examine the expression of WISP3 mRNA. (**F**) Western blot assay was used to detect the WISP3 proteins levels expressed in H1299 and H460 cells treated as (**E**). β-actin was used as a loading control. Quantitative analysis of proteins expression were shown in the histogram. (**G**) H1299 and H460 cells were treated with or without dish-coated fibronectin (10 µg/mL) for 24 h, CHIP assay was performed using HIF-1α antibody to determine the binding site of HIF-1α on WISP3 promoter. (**H**) Luciferase experiment was used to identify the role of fibronectin on the wildtype WISP3 promoter or the mutant one, in which the binding site1 to HIF-1α was mutant. Data were presented by mean ± SD from three independent experiments. *P < 0.05; **P < 0.01 vs. control

The cellular data has manifested that fibronectin could significantly up-regulate the expression of WISP3. We next tried to further confirm the correlation between the expression of fibronectin and WISP3 in adjacent lung cancer tissues by IHC experiments. As shown in Fig. 8A, a positive correlation was observed on the expression of fibronectin and WISP3 in lung cancer tissues. To test whether fibronectin regulated the WISP3 at the transcriptional level, dual luciferase reporter assay was performed and the data shown that fibronectin significantly enhanced the transcription activity of WISP3 promoter in both H460 and H1299 cells (Fig. 8B). Although WISP3 belongs to WISP family, there is almost no research focusing on the underling mechanism of WISP3 expression. Here, the KEGG enrichment analysis of the RNA-seq data indicated that HIF-1α signaling pathway was enriched. We analyzed the potential binding sites for transcriptional factors, and found there is three highly credible binding sites for HIF-1α shown in Fig. 8C. More importantly, fibronectin treatment significantly promoted the expression of HIF-1α protein in both H460 and H1299 cells (Fig. 8D), indeed. Next, siRNA against HIF-1α was used to silence HIF-1α, and the knock-down not only inhibited the expression of WISP3 at mRNA and protein levels alone, but also blocked the role of fibronectin on WISP3 expression (Fig. 8E and F). These findings suggested that fibronectin activated the expression of WISP3 in a HIF-1α-independent manner. Subsequently, CHIP assay was used to confirm which binding site of HIF-1α accounted for the regulation of WISP3 expression. As shown in Fig. 8G, fibronectin treatment induced the binding of HIF-1α protein to site 1. Finally, to further validate this finding, dual luciferase reporter assay was performed. Interestingly, fibronectin could dramatically activate the transcriptional activity of the wildtype WISP3 promoter, while failed in the mutant one (Fig. 8H). Taken together, fibronectin effectively activated the WISP3 expression at transcriptional level via HIF-1α.

## Discussion

Tumor cells secrete proteins function as communication signals in the TME (tumor microenvironment), facilitating the interactions between tumor and/or other recruited cell types to acquire and maintain the cell capability of tumor growth, progression and metastasis [42–44]. Fibronectin, a secreted glycoprotein with high molecular weight in the ECM, which mainly exists in three forms: (i) plasma fibronectin generated by liver cells or endothelial cells, (ii) cellular fibronectin synthesized and secreted by fibroblasts and early mesenchymal cells, and (iii) fetal fibronectin in placental and amniotic tissues [45]. Simultaneously, multitudinous studies have manifested that fibronectin shows distinctive expression in varieties of tumors and plays central roles in carcinogenesis, metastasis, chemoresistance and progression [46]. In NSCLC, fibronectin is overexpressed, and acts as a mitogenic factor. It binds to numerous biological molecules containing integrins, heparin and so on, which activates diverse signaling pathways such as ERK pathway, FAK pathway, mTOR pathways, and WNT/β-catenin pathway down to strengthen cell proliferation, angiogenesis, differentiation and metastasis [16, 18, 47, 48]. Inversely, non-canonical Wnt signaling organized fibronectin matrix [49]. Meanwhile, a study reported that β-catenin, which was originally described as a constituent of the cell adhesion compounds, controlled the expression of the ECM molecule fibronectin [50]. In the present study, fibronectin protein expression is up-regulated in cancer tissues in NSCLC patients, and higher fibronectin expression related to poor prognosis. Interestingly, dish-coated fibronectin promoted cell proliferation, colony formation, migration, invasion, and tube forming activity of NSCLC cells in vitro, mediated the tumor growth, metastasis, angiogenesis in vivo with FAK, WNT/β-catenin, MAPK/ERK signaling pathways activated. Additionally, the treatment of dish-coated fibronectin elevated the expression of EMT markers, N-cadherin and vimentin, concomitant with the improved expression of stemness-related markers, OCT4 and Nanog.

By performing RNA-seq, we initially identified WISP3 was a potential target gene of fibronectin. For decades, increasing researches discovered that WISP3, a gene located on 6q22-6q23, was ectopically expressed in malignant tumors and considered as an oncogene or tumor suppressor gene [25]. It has been certificated that WISP3 was downregulated in BRCA and functioned as a tumor suppressor gene via mediating IGF signaling [51, 52]. Conversely, in colorectal cancer and chondrosarcoma, WISP3 was upregulated [25, 53]. Additionally, WISP3 has been related to both cancer progression and suppression. For instance, knocking WISP3 down lessened cell proliferation and migration in GC (gastric cancer) [26], however, WISP3 accelerated chondrosarcoma migration through upregulating ICAM-1 [54]. In our study, we demonstrated that WISP3 was upregulated in cancer tissues of NSCLC patients and hyper-expression of WISP3 related with a poor prognosis. Furthermore, WISP3 alone could effectively strengthen colony formation, migration, invasion, and angiogenesis in NSCLC cells, and silence of WISP3 sufficiently countervailed the functions of dish-coated fibronectin. The most important is, WISP3 obviously activated the Wnt signaling pathway in turn in NSCLC cells.

Angiogenesis is the growth of new blood vessels. It happens fundamentally during embryogenesis as a crucial procedure for the development of the vascular network [55]. Angiogenic procedures are operated by several cell surface proteins and ECAM (extracellular adhesion molecules) that expressed as angiogenesis maker including Endoglin, VEGF/VEGFR complex, Extra Domain B of fibronectin and so on [56]. In tumors, angiogenesis is an essential process in cancer metastasis and stimulates cancer progression [57]. In our research, dish-coated fibronectin treatment elevated the expression of angiogenesis markers like VEGF, CD31, Tie2 and Ve-cadherin, the similar results were obtained in NSCLC cells with WISP3 overexpressed.

Finally, we tried to elucidate the underling mechanism by which fibronectin could activate the expression of WISP3 in NSCLC, as there is no publication focus on this yet. By analyzing the potential binding sites for transcriptional factor on WISP3 promoter using PROMO, three binding sites for HIF-1α were found. Interestingly, RNA-seq also indicated that HIF-1α signaling pathway was enriched in the DEGs under fibronectin treatment. We firstly confirmed the up-regulation of HIF-1α by dish-coated fibronectin, and further manifested that knock-down of HIF-1α effectively decreased the expression of WISPS and blocked the role of fibronectin on WISP3 expression. By using CHIP assay, site 1 was verified as the site by which HIF-1α indeed binds to WISP3 promoter. At last, by constructing the site 1 mutant WISP3 promoter luciferase reporter plasmid, we ultimately proved that fibronectin activated the transcription of WISP3 via HIF-1α. It is well documented that MAPK/ERK signaling pathways take part in the activation of HIF-1α signaling pathway [58].

**Fig. 9:**
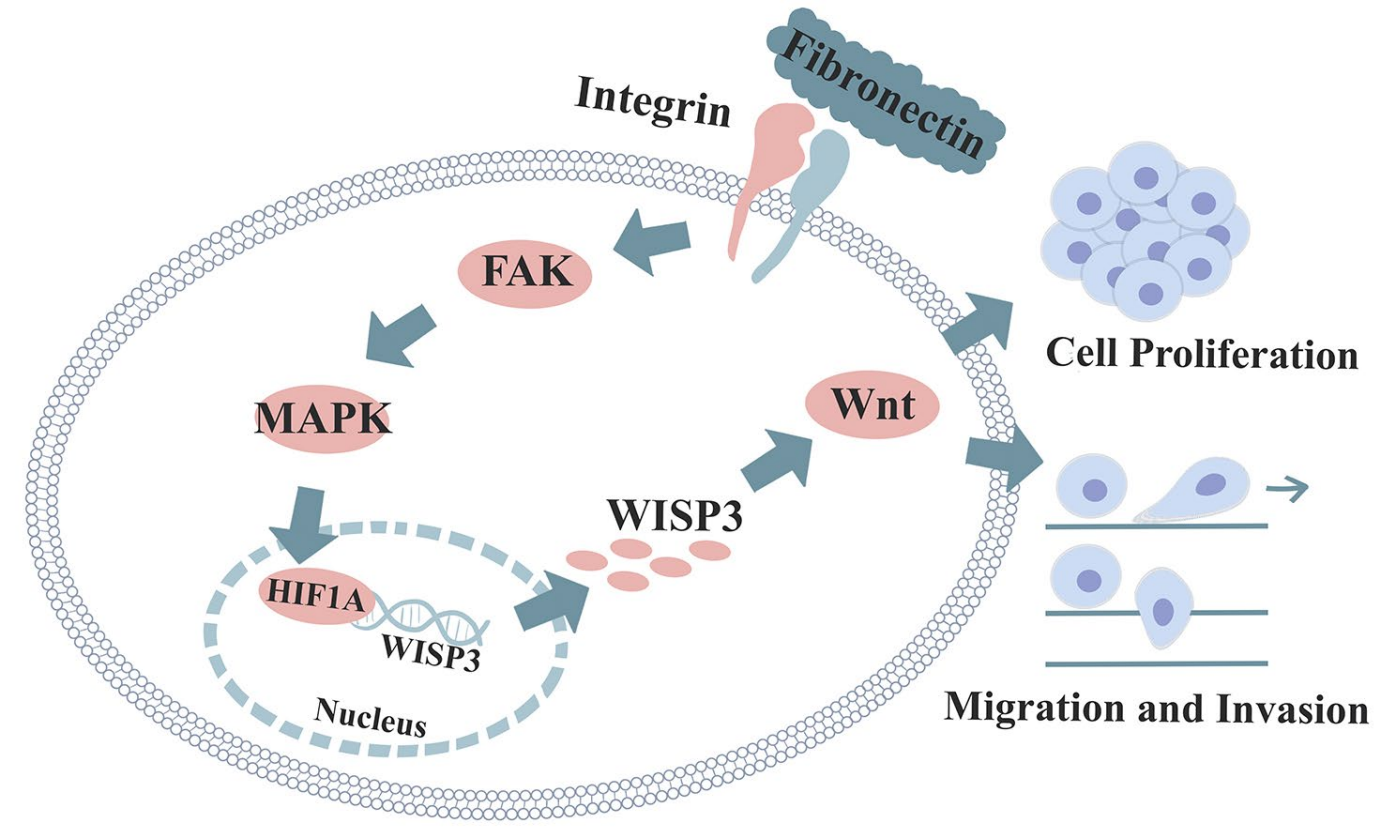
The schematic diagram for how fibronectin functions in NSCLC cells

## Conclusions

Therefore, we hypothesized that exogenous fibronectin binds to integrin on the cell surface to activated downstream signaling pathways such as FAK and MAPK/ERK, which mediated the accumulation of HIF-1α, then, activated HIF-1α and elevated the transcription of WISP3, thus the up-regulation of WISP3 promoted the activation of Wnt signaling pathway, and finally enhanced the tumor growth, metastasis, and angiogenesis in NSCLC (Fig. 9). We believed that more work should be done to further elucidate the relating mechanism for the function of fibronectin in NSCLC. Thereby, dysregulation of fibronectin/WISP3 axis drove the progression of NSCLC, which could be used as a potential prognostic hallmark and a novel therapeutic target.

## Electronic supplementary material

Below is the link to the electronic supplementary material.

Supplementary Material 1: The role of fibronectin on cell migration and invasion in HCC827, H1975 and A549 cells.

Supplementary Material 2

## Data Availability

The datasets used and/or analyzed in the current study are available from the corresponding author on reasonable request.

## Abbreviations

NSCLC: Non‑small cell lung cancer
IHC: Immunohistochemistry
CHIP: Chromatin immunoprecipitation
EMT: Epithelial-Mesenchymal Transition
ECM: Extracellular matrix
ATCC: American Type Culture Collection
DMEM: Dulbecco’s modified eagle medium
PBS: Phosphate Buffer Saline
GO: Gene Ontology
KEGG: Kyoto Encyclopedia of Genes and Genomes
IHC: Immunohistochemistry
