# Supplementary material for: Fibronectin promotes tumor angiogenesis and progression of non-small-cell lung cancer by elevating WISP3 expression via FAK/MAPK/ HIF-1α axis and activating wnt signaling pathway
Source: Exp Hematol Oncol. 2023 Jul 19;12:61. doi: 10.1186/s40164-023-00419-w (PMC10355078; doi:10.1186/s40164-023-00419-w)
Supplement: Supplementary file 1 — Supplementary Material 1: The role of fibronectin on cell migration and invasion in HCC827, H1975 and A549 cells. [file 40164_2023_419_MOESM1_ESM.docx]

**Supplemental Figure 1. Fibronectin exerted contradictory roles on cell moveability of HCC827, H1975, and HA549 cells*.*** (A) The cell migration and invasion of HCC827 cells treated with or without fibronectin (10 μg/mL) for 24h were detected by Transwell assay, and Quantitative analysis of migrated and invaded cells was shown in the histogram. (B) The cell migration of H1975 and A549 cells treated with or without fibronectin (5 or 10 μg/mL) for 24h were detected by Transwell assay, and Quantitative analysis of migrated cells was shown in the histogram. Data was presented by mean ± SD from three independent experiments. *P < 0.05; **P < 0.01 vs. control.
