# Supplementary figures and images for: Fibronectin promotes tumor angiogenesis and progression of non-small-cell lung cancer by elevating WISP3 expression via FAK/MAPK/ HIF-1α axis and activating wnt signaling pathway
Source: Exp Hematol Oncol. 2023 Jul 19;12:61. doi: 10.1186/s40164-023-00419-w (PMC10355078; doi:10.1186/s40164-023-00419-w)

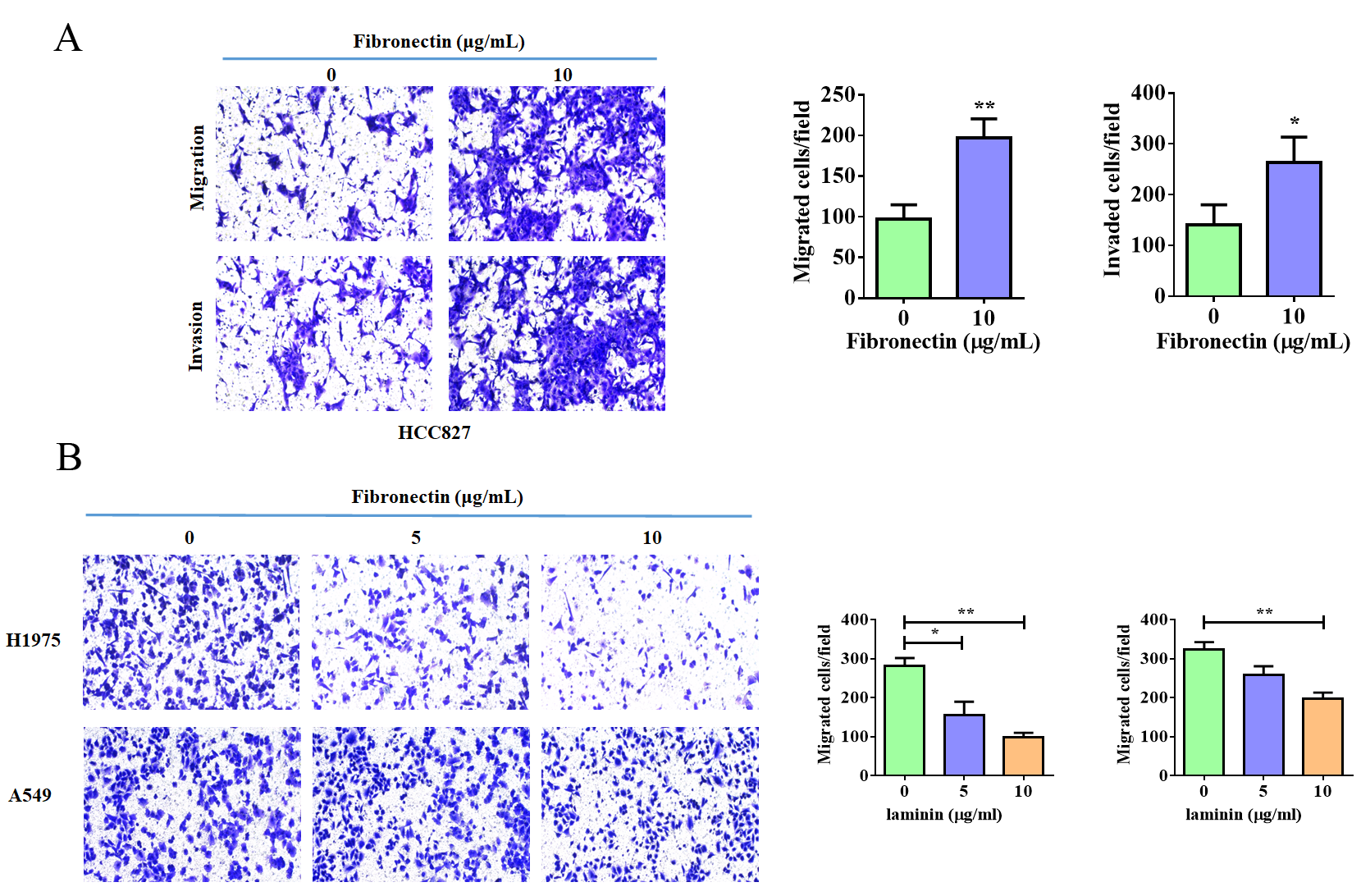

Supplement: Supplementary file 2 — Supplementary Material 2 [file 40164_2023_419_MOESM2_ESM.tif]
